# Exploring the Functional Residues in a Flavin-Binding Fluorescent Protein Using Deep Mutational Scanning

**DOI:** 10.1371/journal.pone.0097817

**Published:** 2014-06-02

**Authors:** HyeonSeok Shin, Yoobok Cho, Dong-hui Choe, Yujin Jeong, Suhyung Cho, Sun Chang Kim, Byung-Kwan Cho

**Affiliations:** 1 Department of Biological Sciences and KAIST Institute for the BioCentury, Korea Advanced Institute of Science and Technology, Daejon, Republic of Korea; 2 Intelligent Synthetic Biology Center, Daejon, Republic of Korea; University of Edinburgh, United Kingdom

## Abstract

Flavin mononucleotide (FMN)-based fluorescent proteins are versatile reporters that can monitor various cellular processes in both aerobic and anaerobic conditions. However, the understanding of the role of individual amino acid residues on the protein function has been limited and has restricted the development of better functional variants. Here we examine the functional amino acid residues of *Escherichia coli* flavin mononucleotide binding fluorescent protein (EcFbFP) using the application of high-throughput sequencing of functional variants, termed deep mutational scanning. The variants were classified into 329 function-retained (FR) and 259 function-loss (FL) mutations, and further the mutational enrichment in each amino acid residues was weighed to find the functionally important residues of EcFbFP. We show that the crucial amino acid residues of EcFbFP lie among the FMN-binding pocket, turns and loops of the protein where conformation changes occur, and spatially clustered residues near the E56-K97 salt bridges. In addition, the mutational sensitivity of the critical residues was confirmed by site-directed mutagenesis. The deep mutational scanning of EcFbFP has demonstrated important implications for constructing better functioning protein variants.

## Introduction

Fluorescent reporter proteins derived from the green fluorescent protein (GFP) and its derivatives have been used to analyze a wide array of cellular processes such as gene regulation, protein localization, and protein interaction [1], [2]. However, GFP-based fluorescent proteins require molecular oxygen for the generation of cyclic tripeptide chromophore, which has been a major drawback in the GFP-based reporter protein applications under conditions with limited oxygen supply. Alternatively, the flavin mononucleotide (FMN)-based fluorescent proteins (FbFPs) can be employed as an *in vivo* reporter system [3]–[5]. Bacterial FbFPs respond to light blue and activate stress-related signaling pathways [6]. Among these FbFPs, *Escherichia coli* FbFP (EcFbFP) which consists of 137 amino acids was engineered from the photoactive LOV (light-oxygen-voltage) domain of *Bacillus subtilis* YtvA, which is a 261-amino acid protein consisting of two functional domains, a core LOV domain and a sulfate transporter and anti-sigma factor (STAS) domain connected by a long linker [7]. In the LOV domain, a metastable covalent bond between a conserved cysteine residue (Cys62) and atom C(4a) of the FMN ring is formed by blue light irradiation (450 nm). In the subsequent reaction where energy is reverted to the ground state, conformational change occurs, which allows emission at the wavelength of 495 nm, wherein the FMN cofactor is non-covalently buried in the LOV domain [8]. Mutation of the cysteine residue to alanine (designated as EcFbFP) increases the fluorescence by tenfold compared to the wild-type LOV domain [7]. Thus, the usage of FMN as a chromophore facilitates EcFbFP to be a promising alternative class of reporter protein capable of expression in both aerobic and anaerobic systems [4]. However, the fitness landscape of individual amino acids that contribute to the florescence of EcFbFP are unknown, except for certain functional residues such as the conserved cysteine and glutamine [7], [8].

The biochemical properties of a protein are coded by its amino acid sequence. This relationship between protein sequence and function is commonly determined by the three-dimensional structure of the protein followed by various biochemical experiments. With explosive growth of genome sequences facilitated by recent advances in sequencing technology, more protein sequences are available compared to the protein three-dimensional structures, and this gap is rapidly widening [9], [10]. Furthermore, conventional methods to unravel critical amino acid residues responsible for specific biochemical functions of a protein are relatively low throughput. Thus, methods for the rapid detection of crucial residues that determine protein functions are needed to accelerate the understanding of the sequence-function relationship.

To this end, high-throughput sequencing technologies have been integrated with mutagenesis of protein-coding sequences in order to elucidate the fitness effect of each amino acid residue [11]. This approach termed deep mutational scanning acquires sequence information from hundreds of thousands of mutants in parallel to fully characterize the sequence-function relationship of a protein of interest. For example, it has been employed to explore functional landscape of amino acid residues in the human Yes-associated protein 65 (hYAP65) [11], human H1N1 influenza [10], and the PDZ domain family [12], [13]. More recently, it has been expanded to assess the effect of individual amino acid residue on the stability of the CH3 domain of human IgG1 [14] and on the appearance of the oseltamivir resistance by the neuraminidase gene of the H1N1 influenza viruses [15]. Although these studies have focused on the affinity of protein domains for their ligands, catalytic function of proteins, such as enzymes, has not been explored by the deep mutational scanning method.

Here we examined the effect of individual amino acid residue on the catalytic function of EcFbFP using random mutagenesis coupled with high-throughput sequencing. Briefly, we exploited PCR-based random mutagenesis to generate protein variants and screened the variant library for the presence or absence of the function of EcFbFP. After 10 cycles of mutagenesis and selection, the accumulated library of function-retained (FR) and function-lost (FL) variants were prepared for high-throughput sequencing with barcoded adaptors for multiplexing. Based on the functional landscape of the amino acid residues in EcFbFP variants, crucial sites that determine EcFbFP function can be categorized into three groups. The first group is located on the direct interaction site of the FMN molecule for facilitating binding, the second group is located on turns and loops of the protein where conformational changes may occur, and the third group is located at spatially clustered residues near the Glu56-Lys97 (E56-K97) salt bridges. Taken together, this approach allows the analysis of thousands of mutations with functional phenotype correlation by single high-throughput sequencing.

## Materials and Methods

### Reagents

All chemicals were purchased from Sigma-Aldrich (St. Louis, USA), unless stated otherwise. Oligonucleotides were purchased from Bioneer (Daejeon, Korea) with standard desalting and were used without further purification. Oligonucleotides used in this study are listed in **Table 1**.

**Table 1:** Oligonucleotides used in this study.

Construct	Primer
EcFbFP_pTrcHis2C	FW: 5′- ATGCCCATGGCGTCGTTCCAGTCGTTCGGC -3′
	RV: 5′- CGATGAATTCTTACTCGAGCAGCTTTTCAT -3′
EcFbFP_ L65F	FW: 5′- GCGCGCTTCTTCCAGGGGAAG -3′
	RV: 5′- CTTCCCCTGGAAGAAGCGCGC -3′
EcFbFP_ R79L	FW: 5′- GACAACATCCTCACCGCGCTG -3′
	RV: 5′- CAGCGCGGTGAGGATGTTGTC -3′
EcFbFP_ N104I	FW: 5′- TGTTCTGGATCGAACTGAAC -3′
	RV: 5′- GTTCAGTTCGATCCAGAACA -3′
EcFbFP_ N104S	FW: 5′- TGTTCTGGAGCGAACTGAAC -3′
	RV: 5′- GTTCAGTTCGCTCCAGAACA -3′

### Bacterial Strains and Cloning

*Escherichia coli* strain TOP10 (Invitrogen, CA, USA) was grown in Luria broth (LB) media at 37°C. The FbFP gene encoding residues 1–137 of the mutant YtvA (C62A) was chemically synthesized and cloned into the pTrcHis2C plasmid (Invitrogen) to yield plasmid pEcFbFP. The pEcFbFP was transformed into *E. coli* TOP10, which were then grown on LB agar plates containing 50 µg/mL ampicillin and 1 mM isopropyl-β-d-thiogalactopyranoside (IPTG). For protein purification, the pEcFbFP gene and the site-direct mutagenesis variants were cloned into pET-28a(+) (Novagen, MA, USA) and transformed into *E. coli* BL21 (DE3).

### Mutant Library Construction

Random mutagenesis was performed using GeneMorph II Random Mutagenesis kit, as per the manufacturer’s instruction (Stratagene, CA, USA). The randomly mutated PCR product was treated with DpnI at 37°C for 1 h to remove the template plasmid and then was digested with EcoRI and NcoI. The digested PCR product was ligated into pTrcHis2C plasmid that was treated with rAPid alkaline phosphatase (Roche, Basel, Switzerland). The ligated plasmids were transformed into *E. coli* strain TOP10, which were grown in LB agar plates with 50 µg/mL ampicillin and 1 mM IPTG at 37°C. The fluorescent colonies and non-fluorescent colonies were visible to naked eye and verified by Gel Doc™ Alexa 488 filter (Bio-Rad, CA, USA). Subsequently, 10^3^ colonies of fluorescent and non-fluorescent *E. coli* were picked and streaked on to LB agar plates containing 1 mM IPTG for the overexpression of EcFbFP overnight to ensure accurate fluorescence selection. Each library was then scraped off the plate, and the plasmids were isolated using DNA-spin Plasmid DNA extraction kit (Intron, NJ, USA). To ensure that the loss of fluorescence in the non-fluorescent colonies is due to mutation, colony PCR was performed to check for self-vector formation in every cycle. The plasmid DNA from fluorescent *E. coli* was used as the template DNA for the next cycle of random mutagenesis.

### Site-directed Mutagenesis

Site directed mutagenesis was performed using the QuikChange site-directed mutagenesis kit (Stratagene) to introduce the single and double mutations into the EcFbFP gene. Briefly, the FbFP gene was amplified from pEcFbFP using *Pfu-X* DNA polymerase (Solgent, Daejeon, Korea), resulting in nicked circular strands of the plasmid. The plasmid was digested using DpnI to remove the residual template DNA and transformed into *E. coli* TOP10 cells by heat shock transformation at 42°C.

### Protein Expression and Purification

The site directed mutagenesis variants were digested using NcoI and XhoI for cloning into the pET-28a(+) vector and transformed to *E. coli* BL21 (DE3). Then, 100 mL LB culture of *E. coli* BL21(DE3) harboring pET-28a(+)_EcFbFP and point mutation variants were cultivated at 37°C for 2–3 h to mid-log phase and induced with 1 mM IPTG at 37°C for 4 h. The cells were harvested by centrifugation and washed with 5 mL lysis buffer (50 mM Tris-HCl (pH 7.5), 200 mM NaCl) and sonicated until the mixture became clear. The clear lysate was collected and incubated in ice with shaking with 320 µL Ni-NTA agarose (Qiagen). Beads were washed with 2 mL wash buffer (50 mM Tris-HCl (pH 7.5), 200 mM NaCl, 50 mM immidazol) and eluted using the elution buffer containing 50 mM Tris-HCl (pH 7.5), 200 mM NaCl, and 250 mM immidazol. Protein concentration of the eluted solution was determined by performing the Bradford assay by measuring the absorbance at 595 nm.

### Fluorescence Measurement

Fluorescent intensity was measured from the purified EcFbFP by Synergy H1 microplate reader (BioTek, VT, USA). The excitation spectrum was determined using the fixed emission wavelength of 495 nm and by modifying the excitation wavelength from 370 nm to 470 nm. The emission spectrum was determined using the fixed excitation wavelength of 450 and by modifying the emission wavelength from 480 nm to 550 nm.

### Sequencing Library Construction and Sequencing

Purified plasmid DNA was quantified using Nanodrop ND-1000 Spectrophotometer (Thermo Scientific, DE, USA) and 1.5 µg of plasmid DNA from each cycle was mixed at the same amount to avoid any bias from a single mutation cycle. The two sets of libraries, fluorescent and non-fluorescent mutants, were prepared for next generation sequencing using the TruSeq DNA Sample Prep Kit v2, according to the manufacturer’s instruction (Illumina, CA, USA). Briefly, 50 µg of mixed plasmids were isolated from each library and fragmented by sonication. The DNA fragments’ ends were repaired, and barcoded adaptors were ligated, as per the manufacturer’s instruction. The ligated product’s size was selected using gel purification, and the product was amplified for 15 cycles. The resulting library was multiplexed in MiSeq v.2 instrument using 50-base-pair single-end reads.

### Sequencing Analysis

Illumina reads were mapped to the pEcFbFP sequence using CLC genomics work bench (insertion cost = 3; deletion cost = 3; similarity fraction = 0.8; and mismatch costs = 2 or 3). The mapped reads were exported to Binary Alignment/Map (BAM) files, and SAMtools were used to extract the reads containing the target region [16]. The first nucleotide of the extracted read was checked for the correct coding frame. Each read was then translated into its amino acid sequence, and mutations were counted using in-house scripts. The sequencing error rate was calculated on the ampicillin resistance gene by dividing the number of mutations in the ampicillin resistance gene over the total number of sequenced reads for FR and FL libraries. Mutations in EcFbFP with lower mutation frequency than the library’s respective sequencing error rate was considered to be a sequencing error and was discarded. The positional effect of each amino acid residue was obtained from the ratio between the sum of mutations in the FL library for each residue and total mutations in the residue.

## Results

### Construction of EcFbFP Variant Libraries

**Figure 1:**
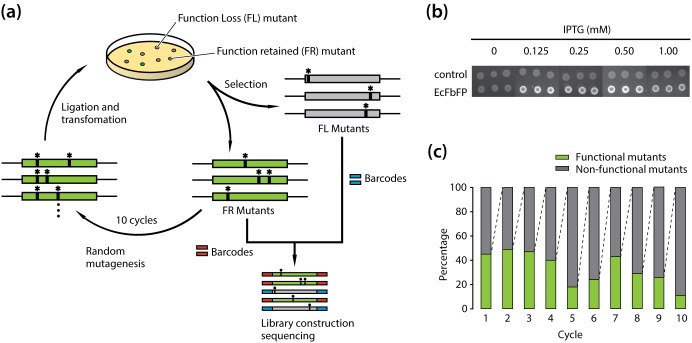
Deep mutational scanning of amino acid residues in EcFbFP. (a) A schematic diagram of the random mutagenesis cycles for functional variants generation and screening. Functional variants were generated using error prone PCR with error rate of 1–6 nucleotide mutations per PCR of EcFbFP and transformed for screening based on the presence or absence of the protein function. For each cycle, mutants showing clear loss of fluorescence and mutants showing retained fluorescence were selected. Function-retained mutants were served as a template DNA for the next mutagenesis cycle. After ten cycles, sequencing libraries of the accumulated function-retained mutants, and the accumulated loss of function mutants were constructed with different barcodes for high-throughput sequencing. (b) IPTG induced the expression of EcFbFP fluorescence with different IPTG concentration plates. Images were recorded under UV illumination. An empty vector without the EcFbFP sequence was used as the control. (c) Ratio of variants that retain function and loose function. For each cycle, ∼1000 EcFbFP mutant colonies were streaked into a Luria broth (LB) agar plate containing 50 µg/mL ampicillin and 1 mM IPTG. The streaked colonies were counted for their functional phenotype by presence or absence of fluorescence.

In spite of its versatility, the development of EcFbFP functional variants was limited due to the lack of information on the role of individual amino acid residues with the protein function. For example, the interaction between a conserved cysteine residue and a FMN molecule in the active site of EcFbFP plays a critical role in absorbing and reacting to blue light [8]. EcFbFP has been engineered to enhance fluorescence intensity and thermostability by site-directed mutagenesis of the residue [17], [18]. However, functional roles of other amino acids in the protein have not been systematically elucidated. To determine the relationship between each amino acid residue and the function of EcFbFP, we used an integrated deep mutational scanning method [10], [11], [13], [14]. It allowed high-throughput and quantitative analysis of functional phenotypes derived from its sequence variation. Briefly, we generated functional variants of EcFbFP using PCR-based random mutagenesis, followed by classifying the variants into two groups (i.e., presence or absence of function). The two variant libraries obtained from ten cycles of mutagenesis were collectively sequenced with barcoded adaptors for multiplexing. Comparative analysis of the amino acid residues between functional and non-functional variants indicated crucial sites responsible for the EcFbFP function (**Figure 1(a)**).

First, *E. coli* cells harboring the wild type EcFbFP gene were grown on solid media, and the *in vivo* fluorescence level was tested. *In vivo* fluorescence generated by the *E. coli* cells was successfully observed only in the presence of isopropyl-β-d-thiogalactopyranoside (IPTG), and fluorescent signal was not detected from *E. coli* cells harboring the plasmid pTrcHis2 only. Any significant changes in fluorescence were not detected by the variation of IPTG concentration (**Figure 1(b)**). Thus, all the variants were tested for fluorescence with the addition of 1 mM of IPTG. This *in vivo* assay allowed us to determine the presence or absence of EcFbFP function by comparing the fluorescent signal of each variant with that of the wild type EcFbFP. Thus, the function loss or function retention of the variants was determined to be due to the intrinsic characteristics of each variant.

### Deep Mutational Scanning of EcFbFP Variants

Next, 10 cycles of random mutagenesis based on error-prone PCR [19] were carried out to incorporate mutations into EcFbFP sequence randomly (**Figure 1(c)**). For each cycle, 200 variants whose function was retained or lost were selected by the presence or absence of fluorescence, respectively, resulting in two libraries of 2×10^3^ variants. To avoid errors in selection, self-vector formation was prevented using DpnI and alkaline phosphatase treatments followed by colony PCR confirmation. Two sets of plasmid DNA were extracted from each cycle and mixed in the same ratio to avoid any bias from a single mutation cycle. The two libraries, FR and FL variants were then subjected to high-throughput sequencing, which were multiplexed to avoid sequencing error from low diversity of nucleotide bases. The high-throughput sequencing resulted in 388,583 and 603,451 raw sequencing reads for FR and FL variants, respectively, with an average length of 50 bp (**Table 2**). Even though the large portion of sequencing reads passed the quality guideline, the base pairs with low quality score were trimmed off for downstream processing using the CLC Genomics Workbench package. After the removal of low quality reads, 98.2% of total reads were uniquely mapped to the EcFBFP sequence. About 1.8% of the sequencing reads were trimmed off due to their short length, low complexity, and overall low quality scores.

**Table 2:** Mapping statistics of FR and FL library.

Library		EcFbFP	Mapped	Total reads
FR library	Number of reads		382,179 (98.25%)	388,583 (100%)
	Average length (bp)		50	50
	Number of bases	4,780	19,147,370 (98.30%)	19,479,487 (100%)
FL library	Number of reads		475,554 (98.14%)	603,451 (100%)
	Average length (bp)		50	50
	Number of bases	4,780	23,829,014 (98.13%)	30,245,033 (100%)

**Figure 2:**
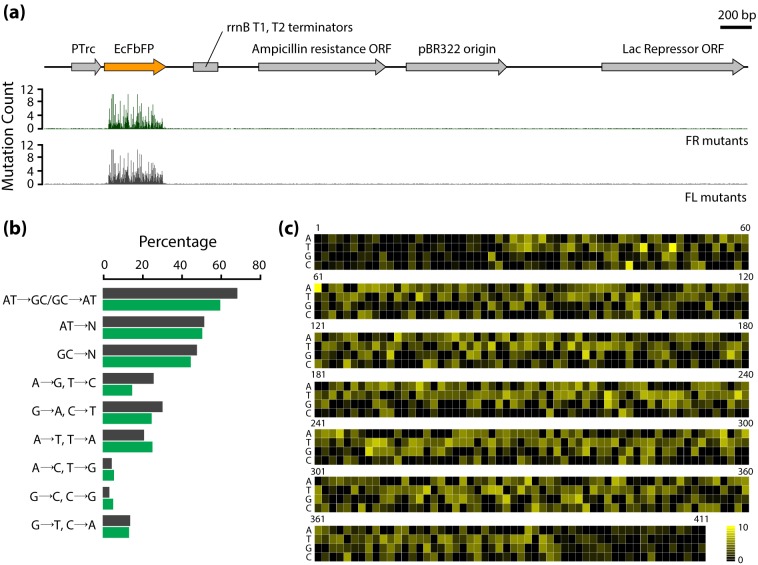
Sequencing results of FR and FL variants. (a) Mapping of the mutation frequency in the whole pTrcHis2CEcFbFP vector. (b) Mutational spectra of induced mutations for each position and each nucleotide is indicated by the black bar. Blue bar indicates the mutational spectra obtained using the mutagenesis kit. (c) Map of the mutation frequencies in EcFbFP at DNA level. Mutations with frequency are shown in yellow.

The mutations in the vector and EcFbFP sequences were first counted from the respective mapped sequences. As predicted, the number of mutations was enriched in EcFbFP sequence and the rest of the vector sequence had negligible non-perfect matches (**Figure 2(a)**). For further validation of the mutation results, we merged the sequencing reads obtained from FR and FL variants and calculated the mutational spectra. Since the mutation spectra show the distribution of mutations occurring at different nucleotides, the accumulated sequencing results of both FR and FL library should be similar to the mutation spectra of the PCR-based mutagenesis method. The mutational spectra exhibited a ratio of 0.9, with slightly higher tendency to create transitions over transversions. As shown in **Figure 2(b)**, the ratio (∼0.68) of transition mutation frequency (ATans/GCans) was consistent with the ratio (∼0.60) described in the manufacturer’s instruction. In addition, the mutation frequency of AT-to-NN was equal to GC-to-NN, indicating that mutational bias is negligible. Taken together, the mutational spectra suggested that most substitutions occurred randomly without positional effect or mutational bias toward any nucleotide (**Figure 2(c)**).

The mapped reads were then converted to amino acid sequences for the detection of single or multiple mutations at protein level. The first nucleotides of the sequencing reads were checked for correct protein-coding frame. If the read is translated into amino acids from the wrong frame, the first one or two nucleotides at the 5′-end were removed accordingly for frame correction. In the case of nucleotides at the 3′-end of each read, the last one or two DNA sequences were removed accordingly for frame correction. After this translational trimming step, we obtained 36,419 (1,521,699 bps) and 29,971 reads (1,155,897 bps) from 36,475 and 30,045 raw mapped reads, indicating that the average depths per nucleotide were 3,702 and 2,812 for the EcFbFP gene in FR and FL libraries, respectively.

Although the error rate of next generation sequencing reads has been investigated [20], we estimated the sequencing error rate based on our experimental data. Since all the mutants were grown on solid media supplemented with ampicillin as a selection marker, no mutations should be detected in the ampicillin resistance gene to preserve its functional integrity. After the translational trimming step, a total of 77,220 (3,490,977 bps) and 99,274 reads (4,473,381 bps) were obtained from 77,268 and 99,330 raw mapped reads for the ampicillin resistance gene in the FR and FL libraries, respectively. The respective average depths were 4,059 and 5,201 for the FR and FL libraries. From the amino acid sequence of the ampicillin resistance gene, we determined the sequencing error rate at the protein level, resulting in 0.13% and 0.14% for FR and FL mutant libraries, respectively (**Table S1**). Mutations in EcFbFP with a frequency less than the calculated sequencing error for each library as well as sequences containing frame shifts were then excluded from further analysis. The average mutation rates for FR and FL libraries at the protein level were 2.57% and 4.38%, respectively, indicating that the number of sequencing errors was negligible compared to the number of mutations in the libraries (**Table S2**).

### Detection of Important Residues that Facilitate EcFbFP Function

**Figure 3:**
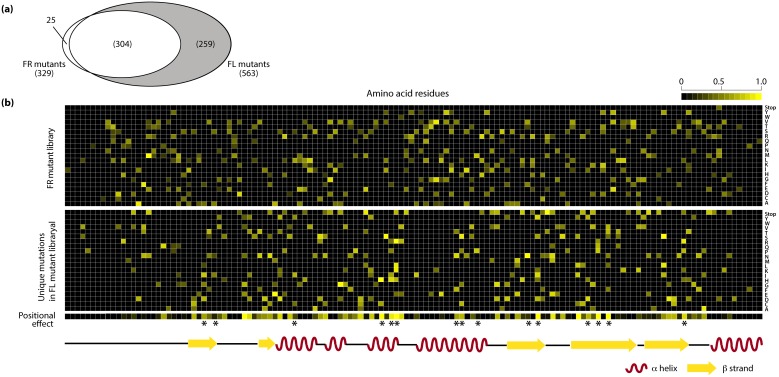
Critical residues determined by mutation frequency. (a) Common and unique mutations in the FL library and FR library. (b) Heatmap of the positions of unique mutations of the FR and FL library with mutation frequency drawn on a scale of 0 to 1. Positions with enriched mutations are shown in yellow. The positional effect bar shows the residues with enriched FL mutations in yellow and residues with enriched FR mutations in black. The asterisks indicate known FMN-binding sites.

Detailed analysis of the amino acid substitutions in variants of FR and FL libraries provided a landscape of the effect of each mutation on the EcFbFP function. All the variants in the FR library were considered to have non-deleterious mutations. On the other hand, each variant in the FL library contains both mutations that have non-deleterious effect and those that cause loss of function, because the variants in FR library were generated by repeated random mutagenesis of FR variant DNAs obtained from each cycle. The number of mutation occurrences in amino acid sequences of variants was 329 and 563 in the FR and FL libraries, respectively (**Figure 3(a)**). Among those, 304 amino acid substitutions were detected in both libraries, which were tolerant to the loss of function. A total of 259 unique mutations were identified from the variants in the FL library, which were expected to be crucial amino acid residues responsible for the EcFbFP function.

In total, the accumulated mutations indicate that each amino acid residue in EcFbFP has an average of 108 mutations; however, the pattern of enriched mutations differ so that some residues are enriched with FR mutations and some residues are enriched with FL mutations (**Table S3**). To analyze this difference in the mutation enrichment pattern, we quantitatively compared the mutation frequency of each amino acid residue in FR and FL variants. The ratio of FL mutations over total mutations (FL and FR mutations) in each residue was calculated. In this study, the ratio of enrichment of FL mutations is referred to as the positional effect. The resulting heatmap of the positional effect indicates that positions tolerant to the deleterious effect on the protein function have a value close to zero and positions sensitive to the deleterious effect on the protein function have a value close to one (**Figure 3(b)**). To further analyze the functionally sensitive positions, we used the 15 FMN-binding sites identified from previous study as a standard to determine the cutoff value for the most sensitive residues of EcFbFP [8]. After applying the cutoff based on the positional effect, we determined 25 functionally sensitive residues, which included ten FMN-binding sites used in the standard.

### Analysis of Functionally Sensitive Residues

**Figure 4:**
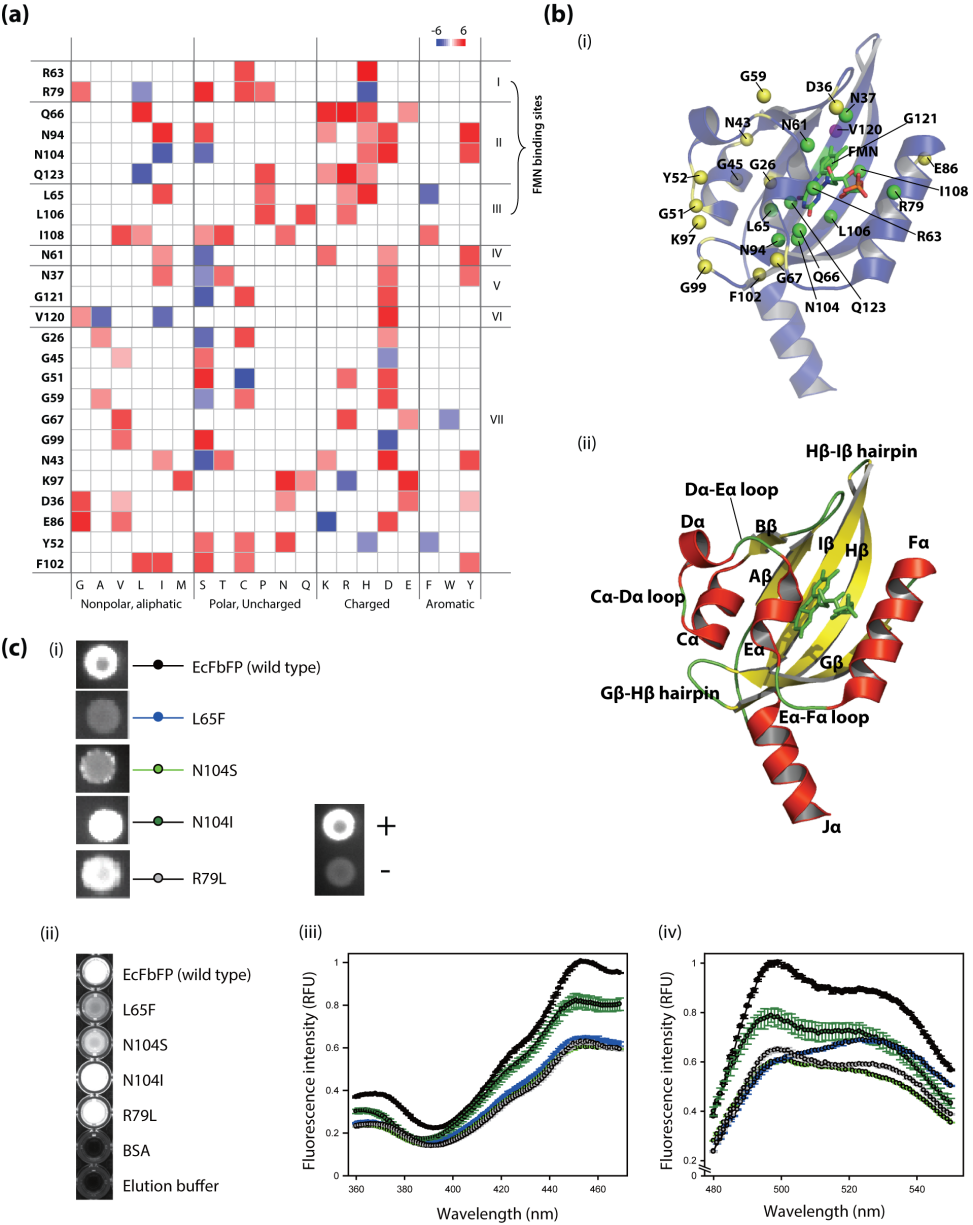
The relationship of critical residues to EcFbFP function. (a) Mapping of the frequency of amino acid changes in 25 sensitive residues of EcFbFP. Known or predicted function of each residue is (I) formation of salt bridge with FMN, (II) formation of hydrogen bond with FMN, (III) hydrophobic contact with FMN, (IV) hydrogen bond with FMN’s ribityl chain, (V) FMN-binding sites predicted by MSDsite, (VI) dimer formation site, and (VII) turns and loops. (b) Structural mapping of the critical residues of EcFbFP generated by the PyMOL software (The PyMOL Molecular Graphics System, Version 1.1r1, Schrödinger, LLC) showing residues 20 to 137 of the LOV domain of YtvA (PDB: 2PR6). (i) Critical residues categorized as FMN-binding sites (I, II, III, and VI) shown in green spheres, dimer forming site (V) shown in violet sphere, and turns and loops are (VII) shown in yellow spheres. To highlight the directional interaction of amino acid side chains of each residue, the spheres in the FMN-binding sites and dimer forming sites show the Cβ atom and the spheres in remaining sites show the Cα atom. (ii) The α-helices is shown in red, β-sheets shown in yellow, loops, hairpins and FMN shown in green. The secondary structures are labeled for clarification. (c) Results of the site-directed mutagenesis of the variants (i) *in vivo* and (ii) in purified form under UV illumination. (iii) The excitation spectrum of purified protein between the wavelength of 360 nm to 470 nm with fixed emission wavelength of 495 nm with fixed emission wavelength of 495 nm, and (iv) *in vitro* measurement of emission spectrum of purified protein between the wavelength of 480 nm to 550 nm with fixed excitation wavelength of 450 nm.

The highly sensitive amino acid residues have higher number of mutations causing deleterious effect on the EcFbFP function. We subcategorized the positions by their functional and structural roles and examined the effects of sequence variations on the protein function. Among the highly sensitive residues, all positions that are located near the FMN-binding pocket play a direct role in stabilizing the cofactor FMN (**Figure 4(a)**). We observed that the positions include two salt-bridge-formation sites with the terminal group of FMN, four sites for hydrogen bonds with the isalloxazine ring of FMN, one site for hydrogen bond with the ribityl chain, and three sites for hydrophobic contact with FMN (**Figure 4(a)**) [8]. All of the sites of EcFbFP that interact with FMN suggest that the mutational scanning analysis detects critical sites in relation to the formation of chromophore.

Comparative analysis of the mutation pattern in each subcategory revealed positions that are non-tolerant to mutation and positions that are fully or partially tolerant to mutations. For example, the four amino acid residues that form hydrogen bonds with the isalloxazine ring of FMN (**Figure 4(a)**) have been changed to a similar group of amino acids. Residue Gln66 and Gln123 had common sequence variation to leucine, lysine, arginine, and histidine; however, the function of Q123L variant was retained while Q66L showed function loss. Similarly, residues Asn94 and Asn104 had five common amino acid variations; however, the functions of N104I and N104S were retained, while the functions of N94I and N94S were lost. This result is consistent with the previous study, where the hydrogen bond formation of FMN to N014 and Q123 was shared [8]. Thus, mutations in N104 or Q123 residues may allow the recovery of the bond formation by the remaining residue, which will still have a hydrogen bond with the FMN isalloxazine ring. These differences in functional pattern allow the identification of residues that are conserved and residues that allow sequence variation. Additionally, two positions (**Figure 4(a)** **, group V**) were located near the FMN-binding pocket and their role was predicted as FMN-binding sites by MSDsite [21]. However, experimental evidence of the interactions with FMN was not found.

To investigate the subset of highly sensitive amino acid residues that did not have any close proximity to the FMN-binding site, we mapped all of the sensitive amino acid residues to the LOV domain of YtvA (**Figure 4(b)**). Residue V120 (**Figure 4(a)** **, group VI**) positioned at the Iβ sheet is the only dimer formation site that shows sensitivity to EcFbFP functions. It is noteworthy that the amino acid substitutions that retain the function of EcFbFP at position 120 (V120A and V120I) are hydrophobic residues. In addition, among nine amino acid residues responsible for the formation of hydrophobic dimer located outside the β-sheets of the strands (Aβ, Bβ, Hβ and Iβ) [8], only residue V120 showed enriched sensitivity to function. To confirm the hydrophobic interaction in homo-dimerization of EcFbFP, we compared the amino acid variation in nine hydrophobic residues located at Aβ, Bβ, Hβ, and Iβ, which mediate the homo-dimerization of YtvA-LOV domain [8]. For each position, the hydrophobic and hydrophilic characteristics of the amino acid substitutions were counted for all amino acids except for glycine, which is aliphatic. In the FL library, four variations are hydrophobic while 149 variations are hydrophilic, which means that 97% of the mutations that cause function loss have been mutated to amino acids with hydrophilic characteristics (**Table 3**). In the FR library, 611 variations were counted as hydrophobic amino acids while 194 variations were hydrophilic amino acids. The highly dimeric state of YtvA-LOV domain has been investigated in previous studies, and our data suggest that the dimer formation of EcFbFP is partially tolerant to sequence variation if the residue is hydrophobic [8].

**Table 3:** Non-deleterious effect of mutations on EcFbFP dimer formation.

Position	FR mutation		FL mutation	
	Hydrophobic	Hydrophilic	Hydrophobic	Hydrophilic
Val25	124	11	0	0
Val27	55	0	0	6
Ile29	44	0	4	28
Tyr41	24	0	0	42
Met111	159	7	0	17
Met113	162	97	0	0
Tyr118	7	12	0	5
Val120	18	0	0	36
Ile122	18	67	0	15
Total	611	194	4	149

The structural positions of the highly sensitive residues clearly show that the remaining subset of sensitive amino acid residues, which is not in any close proximity to the FMN-binding sites or the dimer-formation sites, is distributed along the surface, at loops and turns (**Figure 4(b)** **,** see yellow spheres). Interestingly, among the sensitive positions distributed in turns and loops, the positions located at Gβ-Hβ (GH) hairpin and Cα-Dα (CD) loops are clustered in close proximity to three-dimensional space (**Figure 4(b)**, residues Y52, G51, K97, and G99). This spatial organization is achieved by the salt bridge formed by the residue located at Lys97 and Glu56, which is conserved among many PAS domains (Per; period circadian protein, Arnt; aryl hydrocarbon receptor nuclear translocator protein, Sim; single-minded protein) [22]. We speculated that the salt-bridge must play a critical role in the three-dimensional structure of EcFbFP and examined amino acids that allow function to be retained in the position Lys97 and Glu56. The only variant in residue Lys97 that allows the function to be retained is K97R, which suggests the arginine-glutamic acid salt-bridge formation for the correct three-dimensional structure (**Figure 4(a)**). This is further supported by E56D, the only functional variant found in residue Glu56 that suggests lysine-aspartate salt-bridge formation (**Table S2**). This data suggests that the salt bridge between GH hairpin and Dα must be conserved.

Additionally, the reason for the functional sensitivity of residues Gly51 and Tyr52 is predicted that they have a role in the loop formation between Cα and Dα, since the close spatial proximity of Dα to the residue Lys97 is required for salt-bridge formation. In addition to the structure-stabilizing role of the turns and loops [23], we speculated that their importance in LOV domain is increased by the light activated conformational changes. This prediction is supported by glycine residues, Gly67, Gly59, and Gly26 located within the Eα-Fα loop, the Dα-Eα loop, and the N-terminal linker, respectively. Although EcFbFP does not have a photocyclic function by the mutation C62A [7], the loops are structured with wide angles for flexibility that is provided by the glycine residues. The glycine residues are located in loops surrounding Eα, which is known to be the site of rotation along with Jα. This data correlates with the fact that loops and turns are significant parts of a protein, which stabilize the protein three-dimensional structure by causing changes in the direction of polypeptide chains [24].

### Mutational Sensitivity Test by Site-directed Mutagenesis

To verify the mutational map, positions with FMN-binding sites were selected and tested by site-directed mutagenesis. In this selection, we selected sites that retained their function despite the change in the amino acid characteristics. The *in vivo* detection of fluorescence indicated that all the mutants retained fluorescence as expected by the mutational map (**Figure 4(c)** **, (i)**). However, mutants L65F and N104S showed significant reduction in fluorescence. To quantitatively examine these mutants, we cloned them to an expression vector pET-28a(+), and the protein was purified using Ni-NTA resin for *in vitro* fluorescent assay (**Figure 4(c)** **, (ii)**). The relative fluorescent unit (RFU) indicated a pattern similar to that of the *in vivo* fluorescent assay, except for L65F. L65F showed a very weak fluorescence in the *in vivo* assay; however, the purified L65F showed clear cyan color. In addition, the excitation peak of L65F was measured to be at 524 nm, showing a 27-nm shift from the wild type (**Figure 4(c)** **, (iii), (iv)**). We predicted that these results arise from the dramatic amino acid change of leucine into a bulky aromatic amino acid, phenylalanine. First, this change may cause a steric effect on the stability of the FMN binding to L65F mutant and disturb the bound FMN to be released, causing the shift in emission peak close to the FMN emission peak, which is 530 nm. Second, this change may cause structural changes in the FMN binding pocket, changing the energy state of the molecule and thus the changing the emission wavelength.

## Discussion

We investigated functionally important residues in EcFbFP using random mutagenesis and selection by fluorescence, followed by the mutation analysis based on the high-throughput sequencing. Although the phenotypes were simply classified into FR and FL subgroups, the relationship between 4000 EcFbFP mutants to their phenotypes was determined in a single run of high-throughput sequencing. Using the depth of the enriched mutations in the FR and FL libraries, we characterized the mutations that are sensitive and tolerant to the loss of EcFbFP function. These data were used to calculate the positional effects of mutations on EcFbFP function.

Evaluation of the highly sensitive positions among the FMN-binding sites (**Figure 4(a)**) identified all the residues that display direct bond formation with the FMN cofactor [8]. However, for hydrophobic interaction of FMN with amino acid residues in the EcFbFP active site, six residues (V28, T30, F46, I78, L82 and I92) were defined as highly tolerant to mutation (**Table 4**) [7]. For example, in position L82, the function retained mutation of L82M was highly enriched (mutation count = 197), while average mutation count in a single mutation was 26.5 for FR library. One possible reason is that the docking of FMN to EcFbFP is not highly specific [25]. FMN binds to the LOV domain of YtvA as a chromophore and becomes covalently linked to the conserved cysteine residue on blue-light activation [26], thereby activating the signaling cascade of stress σ response in *B. subtilis* [27]. The structure of the LOV domain in YtvA, thus, contains a strong potential for conformational changes upon activation of the photo-cycle. However, in EcFbFP, only the LOV domain of YtvA is isolated and covalent bond forming cysteine residue, Cys62 is substituted for alanine, which resulted in photo-cycle inhibition [7]. Thus, FMN cofactor is non-covalently bound to the pocket of EcFbFP, and the STAS domain is not linked to the Jα helix [7]. The dynamic potential of EcFbFP for conformational change indicates that the FMN-binding pocket of EcFbFP is not completely rigid and spatially compact.

**Table 4:** Mutation frequency of amino acid residues involved in hydrophobic interaction with FMN.

Position	FR mutations	FL mutations	Positional effect value
Val28	27	45	0.625
Tyr30	53	10	0.159
Phe46	67	31	0.316
Ile78	30	48	0.615
Leu82	197	22	0.101
Ile92	112	40	0.263
Leu65	7	55	0.875
Leu106	0	47	1
Ile108	0	54	1

The homo-dimerization of YtvA-LOV domain is mediated by the hydrophobic residues located at Aβ, Bβ, Hβ, and Iβ [8], and our mutational analysis of FR library agrees with the hydrophobic interaction of homo-dimerization of EcFbFP (**Table 3**). Among the 25 highly sensitive positions of EcFbFP, only a single position, V120 located at the Iβ sheet, was found to be highly sensitive (**Figure 4(b)**). Although we speculated that the homo-dimerization is achieved by a cooperative interaction of hydrophobic residues, our analysis suggests that Iβ sheet may have a higher sensitivity toward homo-dimer stabilization [28]. The added weight of Iβ sheet in homo-dimerization is also supported by the fact that Hβ-Iβ hairpin moves toward the dimer interface upon light absorption [8].

In addition to residues located at the FMN-binding pocket and hydrophobic homo-dimerization sites, highly sensitive residues were located at loops and turns (**Figure 4(b)**). Conformational change in YtvA-LOV is initiated from the FMN-binding pocket and leads to the movement of Eα and Jα helices in a scissor-like motion [8]. Our results indicate that these structural changes are managed through the flexibility of Eα-Fα loops and Dα-Eα loops by the glycine residues at Gly67, Gly59, and Gly26, which support the importance of flexibility in the most dynamically moving structures, EF loops, Eα helix, and Jα helix. On the contrary, residues Tyr52, Gly51, Lys97, and Gly99 spatially clustered near the GH hairpin and CD loops suggest the importance of the structure stabilization role of the salt bridge between K97 and E56. This data further supports the highly conserved salt-bridge in PAS domains, and the only function retaining mutations at these residues are K97R and E56D, which are capable of forming arginine-glutamate salt bridge and lysine-aspartate salt bridge, respectively [22]. We speculated that this salt bridge must be conserved to stabilize the structure in functional form. This notion is further supported by the fact that the salt bridge plays a structure-stabilizing role rather than a role in the receptor activation of the phot1 LOV2 domain in *A. thaliana* [29].

Our method differs from the general screening of positive phenotype by random mutagenesis, as the negative phenotype is the main target. However, our deep mutational scanning data also indicates 329 unique mutations that preserve the function along with the enrichment of each mutation (**Table S2**). Although the variant selection method was based on the presence or absence of function, the analytical approach can be more useful if applied to other mutation induction methods coupled with functional assays. The diverse range of functional variants can be sub-categorized by increase, decrease, retention, and complete loss of function. Consequently, enriched residues for different functional sub-categories can be analyzed using our analytical method that allows comparison of different and conserved enrichment locations between subcategories. Thus, allowing functional weight determination of amino acid residues enables more detailed analysis of protein sequence and function relationship. Another application is the functional analysis of co-existing mutations. The synthetic functional rescue library is a library screened for function gain from loss of function mutants. This method coupled with multiple barcoded adaptors for each cycles, allow the analysis of co-existing mutations similar to the synthetic lethality concept. We suspect that this analysis of multiple mutant behaviors adds another dimension to the investigation of protein function.

Our study determined that the most sensitive residues of EcFbFP lies among the bond-formation residues of the FMN-binding pocket, dynamic conformational changing turns and loops (EF loops, GH hairpin and HI hairpin), and cluster of residues near the E56-K97 salt-bridge formation sites. These results indicate that similar methodology can be applied to find functionally important residues in a wide array of enzymes. Deep mutational scanning was focused on the functionally sensitive residues to find residues that play important roles in EcFbFP as well as the LOV domain. The LOV domain acts as a potent fluorescence reporter in both aerobic and anaerobic system, and its flexibility for incorporation in mammalian system and utility as signaling domain for zinc-finger effector domains indicates that it could emerge as a key reporter system in systems and synthetic biology [3], [30].

### Accession Numbers

The sequencing data were deposited on the NCBI Sequence Read Archive, accession number: PRJNA242983.

## Supporting Information

Table S1 **Mutations in the ampicillin resistance ORF.**
(XLSX)

Table S2 **Mutations in EcFbFP.**
(XLSX)

Table S3 **Unique mutations of EcFbFP in the Function Loss library.**
(XLSX)
